# New Composites LnBDC@AC and CB[6]@AC: From Design toward Selective Adsorption of Methylene Blue or Methyl Orange

**DOI:** 10.1371/journal.pone.0170026

**Published:** 2017-01-20

**Authors:** Guilherme de C. Santos, Amanda L. Barros, Carlos A. F. de Oliveira, Leonis L. da Luz, Fausthon F. da Silva, Grégoire J.-F. Demets, Severino Alves Júnior

**Affiliations:** 1 Departamento de Química Fundamental, Universidade Federal de Pernambuco, Recife, Pernambuco, Brazil; 2 Departamento de Química, Universidade Federal da Paraíba, João Pessoa, Paraíba, Brazil; 3 Departamento de Química, Universidade de São Paulo, Ribeirão Preto, São Paulo, Brazil; The University of Akron, UNITED STATES

## Abstract

New porous composites LnBDC@AC (AC = Activated carbon, Ln = Eu and Gd and BDC = 1,4-benzenedicaboxylate) and CB[6]@AC (CB[6] = Cucurbit[6]uril) were obtained using hydrothermal route. The LnBDC and CB[B] are located inside the pore of the carbon materials as was observed in SEM-EDS, XRPD and FT-IR analysis. Porosimetry analysis showed values typically between AC and LnBDC material, with pore size and surface area, respectively, 29,56 Å and 353.98 m^2^g^-1^ for LnBDC@AC and 35,53 Å and 353.98 m^2^g^-1^ for CB[6]@AC. Both materials showed good absorptive capacity of metil orange (MO) and methylene blue (MB) with selectivity as a function of pH. For acid pH, both materials present selectivity by MB and alkaline pH for MO, with notable performance for CB[6]@AC. Additionally, europium luminescence was used as structural probe to investigate the coordination environment of Eu^3+^ ions in the EuBDC@AC composite after adsorption experiment.

## Introduction

Porous materials with high surface area are shown to be efficient in applications such as fuel cells [1], catalysis [2,3], photovoltaic cells [4], adsorption [5,6] and sensors [7,8]. Activated charcoal (AC) is a common material used in adsorption processes and known for its high porosity and surface area. Due to the presence of many reactive sites in its structure, such as -OH, -COOH, -NH_2_ and other organic groups which can interact with host molecules, AC is one of the most studied adsorbents for the removal of organic pollutants in water [9–11], once; adsorption technology has attracted considerable attention due to its simplicity, high efficiency and low cost [12]. However, this carbonaceous material, for presenting low selectivity, has some limitations in specific applications, such as adsorption of dyes.

An alternative for improving the selectivity is the formation of composites, modifying the pores of the material. This insertion causes changes in the cavitie’s chemical nature and can improve selectivity in the adsorption. The cucurbit[n]uril (CB[n]), for being a rigid macrocycle, symmetrical, composed of hydrophobic and hydrophilic regions [13], has physicochemical properties and biological very peculiar [14], allows a significant capability to interactions with guest molecules [15], forming host-guest complexes with high stability [16]. This macrocyclic structure does not show satisfactory results in the removal of disperse dyes in aqueous solutions [17].

In the literature, we did not find many composites made with cucurbit[6]uril (CB[6]) beyond the polyoxometalate matrix incorporated with this macrocycle, which has been tested as a promising photocatalyst for the dye methyl orange [18,19]. So that it employs CB[6] embedded by a matrix activated charcoal and apply as a potential selective adsorbent material dyes in aqueous solution, it's the first. On the other hand, our group has shown previously crystal growth of coordination polymers (MOFs) within the activated charcoal (AC) pores, forming composites MOF@AC [20]. These composites based on lanthanide succinates have performed favorably in aldicarb adsorption in a biological environment, especially in basic pH. The literature also reports other composites of MOFs with carbonaceous materials as carbon nanotubes (CNTs) [21] and carbon nanofibers (CNF) [22].

In this paper, we report the synthesis in situ and characterization of three new composites constituted of activated carbon (AC), CB[6] and MOFs LnBDC (Ln = Eu e Gd; BDC = 1,4- benzenedicarboxylate). The composites were obtained using hydrothermal synthesis and were characterized by infrared spectroscopy (FT-IR), thermogravimetric analysis (TGA), scanning electron microscopy (SEM-EDS), X-ray powder diffraction (XRPD), porosimetry and luminescence spectroscopy. The dyes adsorption experiments with methyl orange (MO) and methylene blue (MB) were performed at room temperature using UV-VIS detection in different pH values. These materials become important for assigning the activated charcoal a selective character in the adsorptive processes, allowing the selective removal of dyes dissolved in water in pH function.

## Materials and Methods

### Materials

All reagents were used without further purification. Activated carbon and sodium hydroxide (97%) were purchased from Dynamic Ltda. Terephthalic acid (97%) and lanthanide oxides (99.9%) were supplied by Sigma-Aldrich. LnCl_3_·6H_2_O (Ln = Eu^3+^ and Gd^3+^) were obtained by reaction of hydrochloric acid with the corresponding lanthanide oxide. The Cucurbit[6]uril was obtained condensing glycoluril and formaldehyde in HCl 9 mol.L^-1^, as described by Day and co-workers [23] and purified according to the method described by Isaac *et al* [24].

### Syntheses of CB[6]@AC

Cucurbit[6]uril (0.50 g, 0.5 mmol), activated carbon (0.25 g, 50%wt) and 10 mL of water were added in a teflon-lined stainless steel reactor (23 mL). The mixture was heated at 120°C for four days. After this time, the reaction system was cooled to room temperature and the resulting solid (510 mg) was filtered and dried under environmental conditions (Yield 52%, based on the CB[6]).

### Synthesis of LnBDC (Ln_2_(BDC)_3_.(H_2_O)_4_; Ln = Eu^3+^and Gd^3+^)

The synthesis of Na_2_BDC is described by Wanderley *et al* [25]. Initially, 0.50 mmol (0.11 g) of sodium terephthalate and 10 mL of distilled water were mixed in a teflon-lined stainless steel reactor (23 mL), followed by the addition of 1 mmol (0.37 g) EuCl_3_.6H_2_O. The reactor was sealed, heated at 120°C for three days and cooled to room temperature. The resulting solid was filtered, washed with distilled water and air-dried at room temperature for one day (Yield 90%, based on the ligand). This same procedure was used for the GdBDC, replacing the europium chloride for GdCl_3_.6H_2_O (0.37 g, 1 mmol) (Yield 90% based on the ligand).

### Synthesis of LnBDC@AC (Ln = Eu and Gd)

The composites were synthesized using the same procedure of the LnBDC MOFs adding activated carbon in the reaction mixture. Sodium terephthalate (0.5 mmol, 0.11 g) was mixed with 10 mL of water in a Teflon-lined stainless steel reactor (23 mL), followed by the addition of EuCl_3_.6H_2_O (0.37 g, 1 mmol) and 0.24 g of activated charcoal (mass equivalent to 50%wt of the other reagents). The reactor was sealed and the mixture heated to 120°C and held at this temperature for three days. The reaction system was cool to room temperature. The solid obtained was filtered and air-dried at room temperature for one day (mass obtained 364.4 mg). For the gadolinium composite, it was used the same procedure, replacing europium chloride by 1 mmol (0.37 g) of GdCl_3_.6H_2_O (mass obtained 370.1 mg).

### Physical measurements

X-ray analysis (XRPD) were held at room temperature in a diffractometer Shimadzu model XRD-700 diffractometer with K_α_(Cu) 1.54 Å source, step 0.01°, acquisition time of 1 second and 5–50° window. Infrared spectra (4000–400 cm^−1^) were recorded in a Bruker IFS 66 FT-IR spectrophotometer using KBr pallets. The thermogravimetric analyses (TGA) were performed from room temperature to 900°C in a Shimadzu DTG-60H thermal analyzer under a nitrogen flow of 100 mL/min, applying a heating rate of 10°C/min. The SEM images and EDS spectra were obtained using a Shimadzu-SS550 microscope with tungsten filament, voltage of 20 kV, probe 4.0 and working distance between 12 and 17 mm. Nitrogen adsorption measurements were performed at 77 K, using a Micromeritics ASAP 2420 system utilizing BET calculations for surface area. The resulting isotherms were analysed using the BET (BrunauerEmmett-Teller) method. The surface areas, pore volumes and pore sizes determined (see S1–S4 Tables) are the average of two independent measurements. The absorbance at 464 nm and 664 nm for MO and MB respectively were performed in a Shimadzu UV-1800 spectrophotometer. The photoluminescence spectra were obtained in a spectrofluorimeter Horiba-Jobin Yvon Fluorolog-3 with the continuous 450 W xenon lamp and UV xenon flash tube for excitation, double-grating monochromator in the excitation and UV-VIS emission position, single-grating monochromator in the NIR (near infrared) emission position, R928P and H10330A-75 Hamamatsu photomultipliers respectively to UV-VIS and NIR range emissions. All emission spectra were corrected by spectral response of the monochromators and using silicon photodiode reference detector, to monitor and compensate for variation in the xenon lamp output, using typical correction spectra provided by the manufacturer. The zeta potential of the composites were measured in a Malvern Zetasizer Nano analyzer, model ZS90, at pH = 3, 5, 7 and 9.

## Adsorption Experiments

### Calibration curve and stock solution

A stock solution of MB and MO (200 mg/L) was prepared by dissolving MO or MB in deionized water. Calibration curves were obtained for MB and MO monitoring absorbance at wavelength 664 and 464 nm, respectively, only at pH = 7 in the concentration range of 0.40 mg L^-1^ to 14 mg L^-1^. It was obtained an additional calibration curve to MO, at pH = 3, because in the latter case there was change in wavelength absorption. The new absorbance wavelength is 504 nm.

### Adsorption procedure

All solutions used in adsorption experiment and calibration curves for MO and MB were prepared by dilution of the stock solution (200 mg/L) with deionized water. To investigate the dependence of adsorption with pH, we added HCl (0.1 M) or NaOH (0.1 M) solutions to adjust pH value of the mix dyes solutions for pH = 3, 5, 7 and 9. The adsorption experiments were performed by mixing 5 mg of adsorbent (CB[6]@AC or LnBDC@AC; Ln = Eu or Gd) and 10 mL of the dyes mixture 1:1 (MO/MB). The initial concentration of the dyes mixed ([MO]_0_/[MB]_0_) in aqueous solution was set to be 1:1 (40 ppm). The adsorption experiment was conducted in batch with magnetic stirring for 24 h at 30°C and subsequently the suspension was filtered to remove the adsorbent. The concentration of dyes in the supernatant was determinate by UV-VIS spectroscopy. The adsorption capacity (q_e_, mg/g) of the adsorbent can be calculated by applying Eq 1, where C_0_ and C_e_ represent the initial and equilibrium dye concentrations of aqueous solution (mg/L), V is the volume of the solution (L), and m is the mass of the adsorbent (g). This equation is commonly used in the literature. [26,27].

$$q_{e}=\frac{\left(C_{0}-C_{e}\right)V}{m}$$ (1)

## Results and Discussion

### SEM-EDS analysis

**Fig 1:**
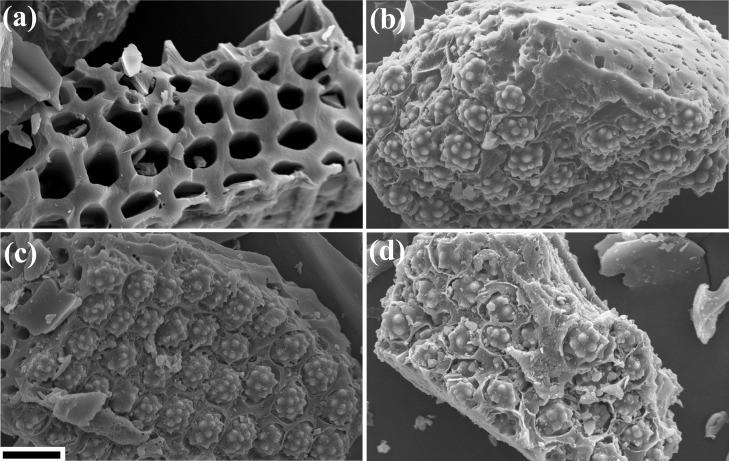
SEM image of AC (a) EuBDC@AC (b) GdBDC@AC (c) and CB[6]@AC (d). The black bar in the figure corresponds to 15 μm.

**Fig 2:**
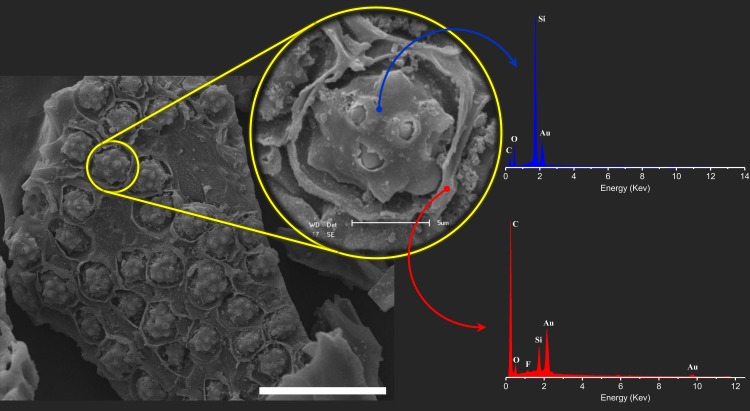
SEM micrograph and EDS spectra inside (blue spectrum) and outside (red spectrum) of the pore from CB[6]@AC composite. The white bar of the figure corresponds to 30 μm.

The distribution of LnBDC and CB[6] in the LnBDC@AC and CB[6]@AC, respectively was determined by scanning electron microscopy and energy dispersive spectroscopy (SEM-EDS). The images show the pores of activated charcoal empty (Fig 1A) and filled by LnBDC (Fig 1B and 1C) and CB[6] (Figs 1D and 2). These results are similar to activated charcoal in the Ln-succinate@AC composite (Ln = Eu and Tb), already reported by our research group in a previous work, also using hydrothermal method [20]. Thus, this confirms the incorporation of LnBDC MOFs and the CB[6] inside the AC pores. However, the insertion of macrocyclic molecule such as cucurbit[n]uril in AC pores, until now, was not been reported in the literature.

EDS analysis inside and outside the pore for LnBDC@AC (S1 and S2 Figs) and CB[6]@AC (Fig 2) composites show distinct composition in these regions. All EDS spectra outside the pores have similar profiles, containing signals of C, O, Si and Au. The Au signals are arising from metallization procedure in the sample preparation and others signals are correlated to AC, whereby Si signal is arising from AC source, such as rice husks, bamboo, etc., as reported for several articles [28–30]. The EDS spectra inside the pore in the LnBDC@AC composite reveal the presence of lanthanides ions (Eu^3+^ or Gd^3+^), C and O confirming the presence of MOFs. Furthermore, these spectra show that the pores have a larger amount of oxygen in detriment of EDS spectra outside of pore (S1 and S2 Figs). The CB[6]@AC composite shows a similar behaviour for EDS spectra inside and outside. However, the signal from nitrogen of CB[6] has not been observed.

### X-ray powder diffraction

**Fig 3:**
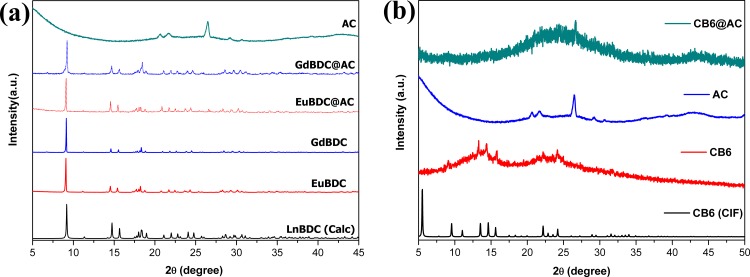
XRPD of AC, LnBDC, LnBDC@AC and LnBDC calculated from the crystal structure [29]. (Ln = Eu and Gd) (a), and of AC, CB[6]@AC, CB[6] and calculated from the crystal structure.

The experimental XRPD pattern of as synthesized LnBDC (Fig 3) is in good agreement with calculated for TbBDC, firstly reported by Yaghi *et al* [31]. Wherefore, the EuBDC and GdBDC obtained are isostructural to this reported structure. This 3D coordination polymer crystallize in the triclinic system and P1 space group, with the lanthanide ions coordinated to six oxygens from BDC^2-^ anions and two water ligands to give an eight-coordinated Ln(III) center [31]. The LnBDC@AC powder patters are in agreement with the free LnBDC MOFs, which indicates the same crystalline structure was formed inside the charcoal pores.

Although the CB[6] may present some crystalline phases [32], in this work it was synthesised in semicrystalline form (see Fig 3B). PXRD pattern of CB[6]@AC composite shows typical amorphic bands of AC (Fig 3B).

### Vibrational Spectroscopy and Thermal Analysis

Infrared spectra of LnBDC (Ln = Eu^3+^ and Gd^3+^) and their respective composites are similar (S3 Fig), showing the expected strong characteristic absorptions for the symmetric and asymmetric vibrations of carboxylate group from the BDC ligand (1610–1550 and 1420–1335 cm^-1^). The broad band centred at 3460 cm^-1^ corresponding to asymmetric and asymmetrical stretching of the water molecules. These infrared spectra showed no absorptions of any protonated BDC (1715–1680 cm^-1^) or their sodium salt [25], confirming the formation of the compound. These results are in good agreement with the XRPD experimental data.

The IR spectrum of CB[6]AC composite shows same vibrational modes of the free CB[6], confirming their presence in the composite (S4 Fig). The peaks at 1726 cm^-1^, 1325 cm^-1^, 1372 cm^-1^, and 1478 cm^-1^, correspond to *v*_*C = O*_, *v*_*C−N*_ and *v*_*N−H*_ stretching, respectively. Signals of vibrational modes of AC have not been observed in the infrared spectra of CB[6]@AC composite because these signals are covered by the vibrational bands of the CB[6] molecule. In addition, O-H stretching from adsorbed water molecules between 3300 and 3700 cm^-1^ are clearly visible.

**Fig 4:**
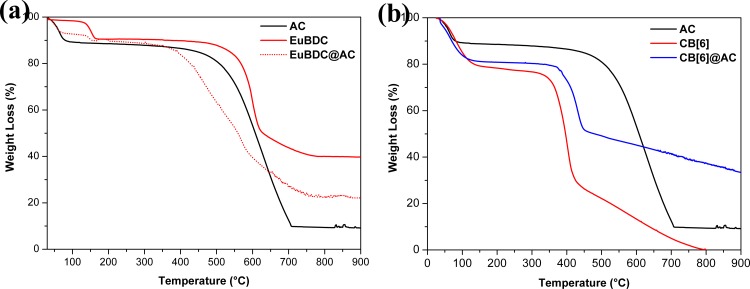
TGA analysis of AC, EuBDC and EuBDC@AC (a), and of AC, CB[6] and CB[6]@AC (b).

Thermal analysis of AC (Fig 4A and 4B; solid black lines) shows two well defined mass loss events. The first event corresponds to the loss of adsorbed water molecules and the second one (near of 400°C) is assigned to the carbonaceous materials decomposition. The thermal decomposition of EuBDC and EuBDC@AC are similar (Fig 4A; solid and dotted red lines). The loss of hydrated and coordinated water molecules occurs up to 180°C, in both materials. The total thermal decomposition in LnBDC starts near 500°C, while for the EuBDC@AC, this process begins near 350°C, indicating a lower thermal stability compared to free MOF and AC. Similar results for the GdBDC@AC were found (S5 Fig). On the other hand, CB[6]@AC composite shows higher thermal stability compared to free CB[6] (see Fig 4B). For CB[6] and the respective composite, a initial mass loss was observed, corresponding to adsorbed water molecules, also confirmed in the vibrational spectra. The subsequent mass losses are correlated with organic decomposition.

### Porosimetry

**Fig 5:**
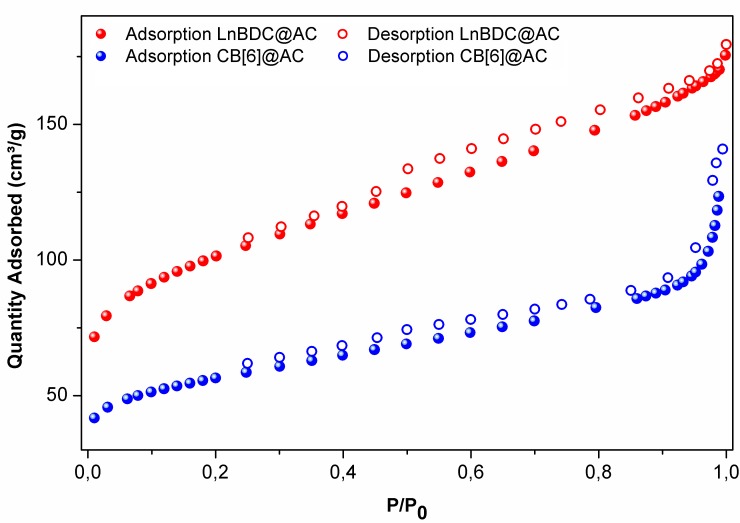
Adsorption isotherms of CB[6]@AC (a) and EuBDC@AC (b).

The adsorption isotherms of N_2_ at 77K of AC, EuBDC@AC and CB[6]@AC materials, and their surface areas, pore volume and pore size data are shown in Fig 5, S6 Fig and S1 Table. For the synthesized composites, the correlation coefficient in the BET surface area was 0.9998 to both composites. For the activated carbon this parameter is 0.9996, indicating types of isotherms favourable to adsorptive processes since this coefficient should be greater than zero and less than one [31]. The commercial AC, used as platform to development of all composites materials here presented, exhibits the typical isotherm shape (type II) of meso/macroporous carbonaceous materials (S6 Fig) [33–35]. Similarly to the AC, the EuBDC@AC composite maintains the type II isotherm (see Fig 5), due to EuBCD to be a non-porous material and display same isotherm shape [36]. However, the EuBDC@AC presents an intense decreasing in the surface area and pore volume (S1 Table), related to the physical occupation of the AC pores. On the other hand, the isotherm shape of CB[6]@AC composite could be categorized as type IV, indicating the presence of micro and mesopores. The microporous behaviour has been assigned to CB[6] inside of pore of the AC, however, the BET model has less applicability to micropores materials [37], leading to small value of surface area (196 m^2^ g^-1^) and pore volume (0.17 cm³ g^-1^) displayed for CB[6]@AC in detriment to AC and EuBDC@AC (S1 Table).

A increasing in the pore diameters were observed for all composite, relative to the AC material, especially for CB[6]@AC material. Probably, this behaviour is intrinsically correlated to microporosity of cucurbituril molecules. This effect was less pronounced in the LnMOF-based composites.

### Adsorption properties

Batch adsorption tests with composites were preformed to evaluate the selectivity in the sorption of methyl orange (MO) and methylene blue (MB) dyes as a function of pH, varying from 3 to 9. The CB[6]@AC composite shows the best results in terms of selectivity. At pH 3, CB[6]@AC adsorbs preferentially MO. However, by increasing pH this behaviour was modified step by step, and from pH 7 was inverted, passing the MB be adsorbed preferentially. This behaviour may be occurring due to electrostatic interaction between the adsorbent and the dyes. In this case, the attraction force between the composite and MB, which is a cationic dye, increases in higher pH values. The zeta potential of the CB[6]@AC (S2 Table) was measured in ranges between 16 mV (at pH 3) to -30 mV (at pH 9), and the results are in good agreement with the higher adsorption for the cationic dye in alkaline medium.

Both dyes have accessible terminal groups = N^+^(CH_3_)_2_ in their structures, which can bind with the carbonyls of CB[6] by interactions of the type ion dipole. Because the MO is a smaller molecule than the MB, lower steric impediments may be associated within such connections; beyond which, its turning point occurs at pH 3, range is that nitrogen is protonated. Already at higher pH values, by this dye loses its positive charge and remain only as charged grouping the SO_3_^-^, reduces considerably the chances of interaction with CB[6], unlike the MB, which interacts with the macrocycle horizontally same at higher pHs because it does not fail to be cationic [38,39]. The MO in turn is an anionic dye and will then be attracted by the adsorbent at this pH and it is related to the positive zeta potential observed in acidic medium.

**Fig 6:**
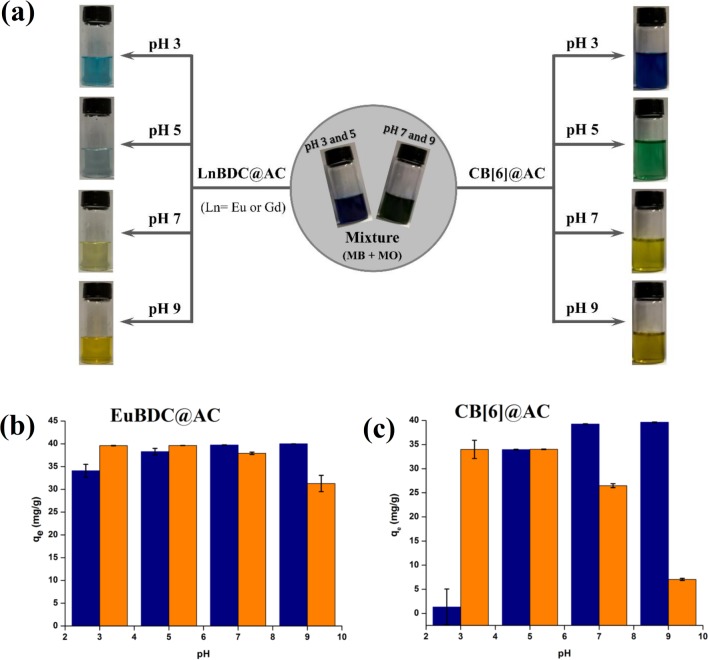
(a) Schematic representation of dyes mixer (MO + MB) adsorption in LnBDC@AC (left) and CB[6]@AC (right) showing the final placing of the supernatant, and q_e_ variation of (b) LnBDC@AC and (c) CB[6]@AC for mixture of dyes in pH function.

For the system EuBDC@AC, we observe a previous similar behaviour, but with a less pronounced selectivity, a possible interpretation for this is in the fact that the system EuBDC, the oxygen atoms from the ligand should meet coordinated to the Eu^3+^, making the composite is less affected by changes in pH. Again, the selectivity behaviour in agreement with the zeta potential measured (S3 Table). Visually comparing the solutions colours after adsorption with composites and pure AC the selectivity is clear. The visual appearance for the composite with MOFs and charcoal alone are shown in the Fig 6 and S7 Fig.

The adsorptive capacities of the composites were also obtained in each dye individually and also of pure LnBDC, pure CB[6] and pure AC for both the dye mixture and each dye individually (S8–S12 Figs). The pure activated carbon has a high adsorption capacity than both MB and MO, as expected. However, when analyzing their behaviour in the presence of the dye mixture, similar adsorptive capacities for MB and MO were observed, therefore, there is no selectivity in the adsorption. The composites have values of q_e_ for each dye individually lower than that of activated carbon, but, as already reported, when analyzing the dye mixture; these systems have selectivity in the adsorption of the dyes as a function of pH. The pure LnBDC and CB[6] also exhibit selectivity, but in some cases they show less adsorptive capacity than composites. Composites in turn have higher adsorption capacity, due to the carbon, along with the selectivity of the pure compounds.

**Fig 7:**
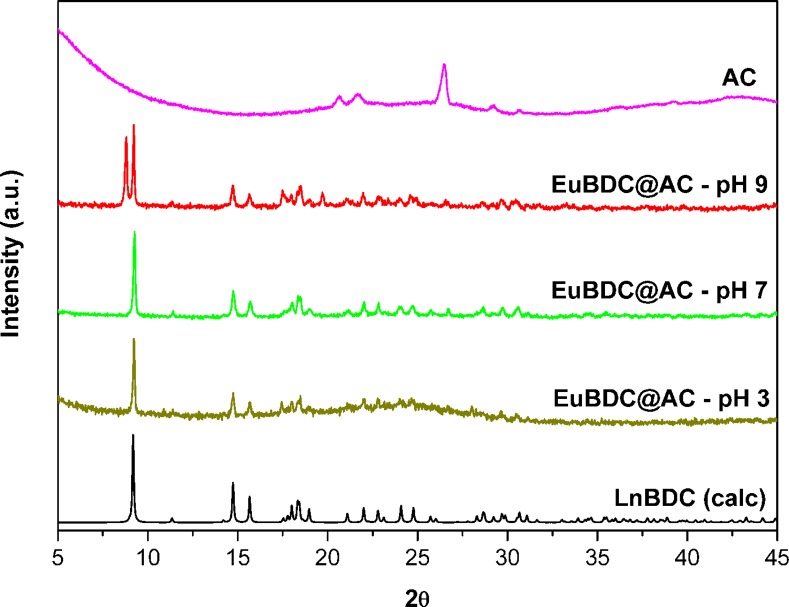
XRPD of AC, EuBDC@AC at pH = 3, 7 and 9, and LnBDC calculated [29].

Powder diffraction patterns of the EuBDC@AC composite after adsorption experiments at pH = 3, 5, 7 and 9 are shown in the Fig 7. No significant changes were observed in the diffraction pattern of the composite at pH = 5, 7 and 9. But, at pH 3, an amorphic broad band was observed, related to acidic degradation of MOF in the EuBDC@AC composite.

### Adsorption isotherms

The adsorption isotherms were performed at pH 5, under agitation for 24 hours similarly to the other adsorption tests. Adsorption isotherm is important to describe adsorption behaviour of different adsorbents. There are several isotherm equations available for analyzing experimental sorption equilibrium parameters, the most common being the Langmuir and Freundlich models [40].

The Langmuir adsorption isotherm has vastly been used to appreciate the performance of different adsorbents. Its model assumes monolayer adsorption, and it refers to homogeneous adsorption. [41] The linear form of the Langmuir isotherm can be represented by Eq 2:

$$\frac{C_{e}}{q_{e}}=\frac{C_{e}}{Q_{0}}+\frac{1}{Q_{0}b}$$ (2)

where C_e_ is equilibrium concentration of adsorbate (mg/L); q_e_ is adsorption capacity (mg/g); Q_0_ is Langmuir constant (maximum adsorption capacity) (mg/g) and b is Langmuir constant (L/mg).

The Freundlich isotherm model, which is widely used to describe heterogeneous systems, it can be applied to multilayer adsorption, with nonuniform distributions. [42] The linear form Freundlich isotherm is given by the following Eq 3:

$$\ln q_{e}=\ln k_{f}+\left(\frac{1}{n}\right)\ln C_{e}$$ (3)

where K_F_ ((mg/g)(L/mg)^1/n^) is roughly an indicator of the adsorption capacity and (1/n) of the adsorption intensity. Value of n > 1 represents a favorable adsorption condition. [43]

S13–S20 Figs show the adsorption isotherms plots of the Langmuir and Freundlich equation. S4 Table summarizes the parameters of the obtained isotherms. The data obtained indicate a better fit to the Langmuir model, which is an indicative of monolayer adsorption of the systems. The data obtained indicate a better fit to the Langmuir model, except for the system CB[6]@AC with MO. The fit to the Langmuir model is indicative of the monolayer adsorption of the systems.

Systems with the LnBDC@AC composite present higher values of Q_0_ for MB adsorption. This was already expected due to the fact that pure LnBDC and AC have higher adsorptive capacities for MB than for MO. For the CB[6]@AC system more advanced studies are needed.

### Photoluminescent properties

**Fig 8:**
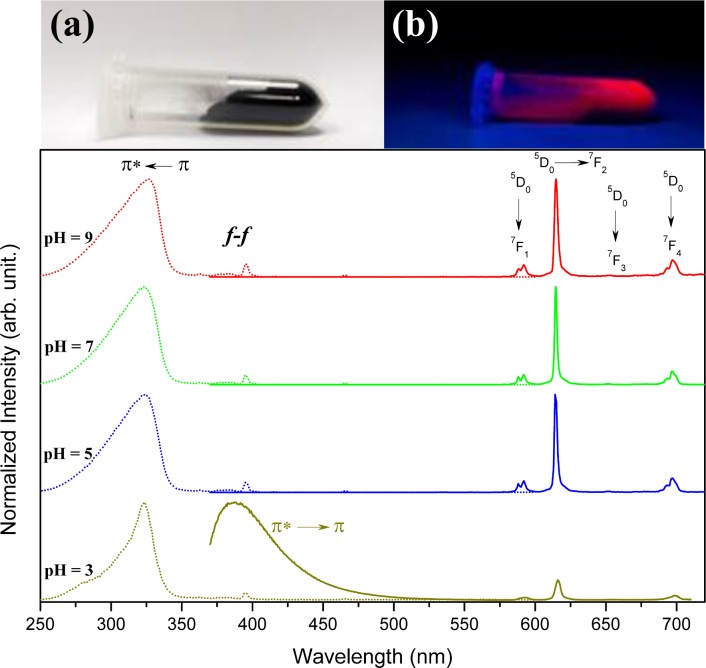
Excitation (dotted lines; λ_Em_ = 615 nm) and emission (solid lines; λ_Ex_ = 325 nm) spectra of EuBDC@AC composite after MO adsorption at pH = 3, 5, 7 and 9.

**Fig 9:**
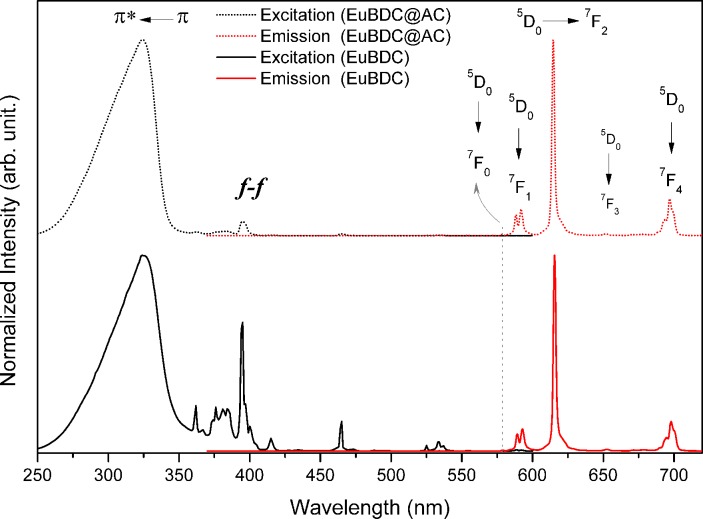
Excitation (black lines; λ_Em_ = 615 nm) and emission (red lines; λ_Ex_ = 325 nm) spectra of EuBDC (solid line) and EuBDC@AC (doted line).

Images of the EuBDC@AC composite under white (Fig 8A) and UV (Fig 8B) light illumination show the natural red luminescence of this materials related to the Eu^3+^ ions. The Photoluminescence properties of EuBDC@AC composite were investigated at room temperature before (Fig 9) and after (Fig 8C) the adsorption experiments in different pH values. The excitation (dotted lines of Fig 8) and emission (solid lines of Fig 8) spectra were acquired upon monitoring emission at 615 nm and exciting at 325 nm, respectively, for these composites before and after adsorption experiment. The europium ion was used as a spectroscopic probe for the symmetry integrity investigation of the first coordination sphere. Once the relative intensities and the maximum Stark splitting of ^7^F_J_ (J = 0, 1, 2, 3 and 4) levels, which are dependent upon the extent in which the level degeneracy (2J+1) is removed by the symmetry of the first coordination sphere [44,45].

Thus, small changes in the structure and composition, due to acidic degradation and/or interaction to europium ion with adsorbate, can be detected. The emission spectra of EuBDC and EuBDC@AC composite (see Fig 9 and S21 Fig), display the same spectral profiles characteristic of the Eu^3+ 5^D_0_→^7^F_J_ (J = 1–4) transitions consistent with pseudo C_4_ symmetry environment around europium ion [46].

This indicates no changes in the Eu^3+^ local environment with the confinement of EuBDC into the AC pore as well as reported by our group for lanthanide succinates/AC composites [20] as above discussed. The excitations and emissions spectra after the dye adsorption (Fig 8C) show identical spectral profiles for free EuBDC MOF, thus, no structural alterations or significant defects were caused during adsorption experiments at pH 5, 7 and 9. On the other hand, the spectral profile after adsorption experiment at the pH 3 shows a significant change with the appearance to an emission broad band between 370–500 nm assigned to MO adsorbed. This behaviour suggests the acidic degradation of MOF resulting in the crystallinity loss and modifications on the Eu^3+^ coordination environment. All these results are in agreement with XRPD experimental data.

## Conclusions

We demonstrate the synthesis of the new composites based on the insertion of LnBDC (Ln = Eu and Gd) MOFs and CB[6] molecules inside activated charcoal pores. These systems showed selective pH dependent due to specific interactions between dyes (MO and MB) and the surface of the adsorbents (LnBDC@AC and CB[6]@AC). The specific adsorption may be tunable by pH range selected, since the adsorption of MO is favored by acid pH and the adsorption of MB by the basic pH. The CB[6]@AC composite shows higher selectivity for the cationic-MB in the equimolar mixtures, accounting for better results in the acidic pH. The LnBDC@AC composite presents smaller specificity of adsorption as a function of pH tuning, however, exhibits better potential to adsorb large amounts of both, with rapid kinetics. Thus, these composite materials play an important role in adsorbing and removing dyes from contaminated water, as well as dyes separation.

## Supporting Information

S1 Fig EDS spectra of EuBDC@AC inside (a) and outside of porous (b).
(TIF)

S2 Fig EDS spectra of GdBDC@AC inside (a) and outside of porous (b).
(TIF)

S3 Fig FTIR spectra of AC, LnBDC and LnBDC@AC (Ln = Eu and Gd). (TIF)

S4 Fig FTIR spectra of AC, CB[6] and CB[6]@AC. (TIF)

S5 Fig TGA analysis of AC, GdBDC and GdBDC@AC. (TIF)

S6 Fig Adsorption isotherms of AC. (TIF)

S7 Fig Schematic representation of dyes mixer (MO + MB) adsorption in AC showing the final placing of the column brown), for the dye mixture (Column cyano and yellow). (TIFF)

S8 Fig Adsorptive capacities of the composite LnBDC@AC, for individual dyes, Methylene Blue (Column blue), Methyl Orange (Column brown), for the dye mixture (Column cyano and yellow). (TIF)

S9 Fig Adsorptive capacities of pure CB[6], for individual dyes, Methylene Blue (Column blue), Methyl Orange (Column brown), for the dye mixture (Column cyano and yellow). (TIF)

S10 Fig Adsorptive capacities of pure CB[6], for individual dyes, Methylene Blue (Column blue), Methyl Orange (Column brown), for the dye mixture (Column cyan and yellow). (TIF)

S11 Fig Adsorptive capacities of the composite CB[6]@AC, for individual dyes, Methylene Blue (Column blue), Methyl Orange (Column brown), for the dye mixture (Column cyano and yellow). (TIF)

S12 Fig Adsorptive capacities of pure AC, for individual dyes, Methylene Blue (Column blue), Methyl Orange (Column brown), for the dye mixture (Column cyano and yellow). (TIF)

S13 Fig Langmuir adsorption isotherm of the composite LnBDC@AC with methylene blue. (TIF)

S14 Fig Langmuir adsorption isotherm of the composite LnBDC@AC with methyl orange. (TIF)

S15 Fig Langmuir adsorption isotherm of the composite CB[6]@AC with methylene blue. (TIF)

S16 Fig Langmuir adsorption isotherm of the composite CB[6]@AC with methyl orange. (TIF)

S17 Fig Freundlich adsorption isotherm of the composite LnBDC@AC with methylene blue. (TIF)

S18 Fig Freundlich adsorption isotherm of the composite LnBDC@AC with methyl orange. (TIF)

S19 Fig Freundlich adsorption isotherm of the composite CB[6]@AC with methylene blue. (TIF)

S20 Fig Freundlich adsorption isotherm of the composite CB[6]@AC with methyl orange. (TIF)

S21 Fig Excitation (λ_Em_ = 615 nm) and emission (λ_Ex_ = 325 nm) spectra of EuBDC. (TIF)

S22 Fig Excitation (black line) and emission (blue line) spectra of MO. (TIFF)

S23 Fig Calibrations curves of methylene blue in pH 7 (a), methyl orange in pH 7 (b) and methyl orange pH 3(c).
(TIFF)

S1 Table Experimental parameters of the porosimetry. (XLSX)

S2 Table Zeta potential of CB[6] and CB[6]@AC. (XLSX)

S3 Table Zeta potential of MOF (LnBDC) and LnBDC@AC. (XLSX)

S4 Table Adsorption parameters of Langmuir and Freundlich isotherms. (XLSX)
