# Supplementary figures and images for: New Composites LnBDC@AC and CB[6]@AC: From Design toward Selective Adsorption of Methylene Blue or Methyl Orange
Source: PLoS One. 2017 Jan 20;12(1):e0170026. doi: 10.1371/journal.pone.0170026 (PMC5249156; doi:10.1371/journal.pone.0170026)

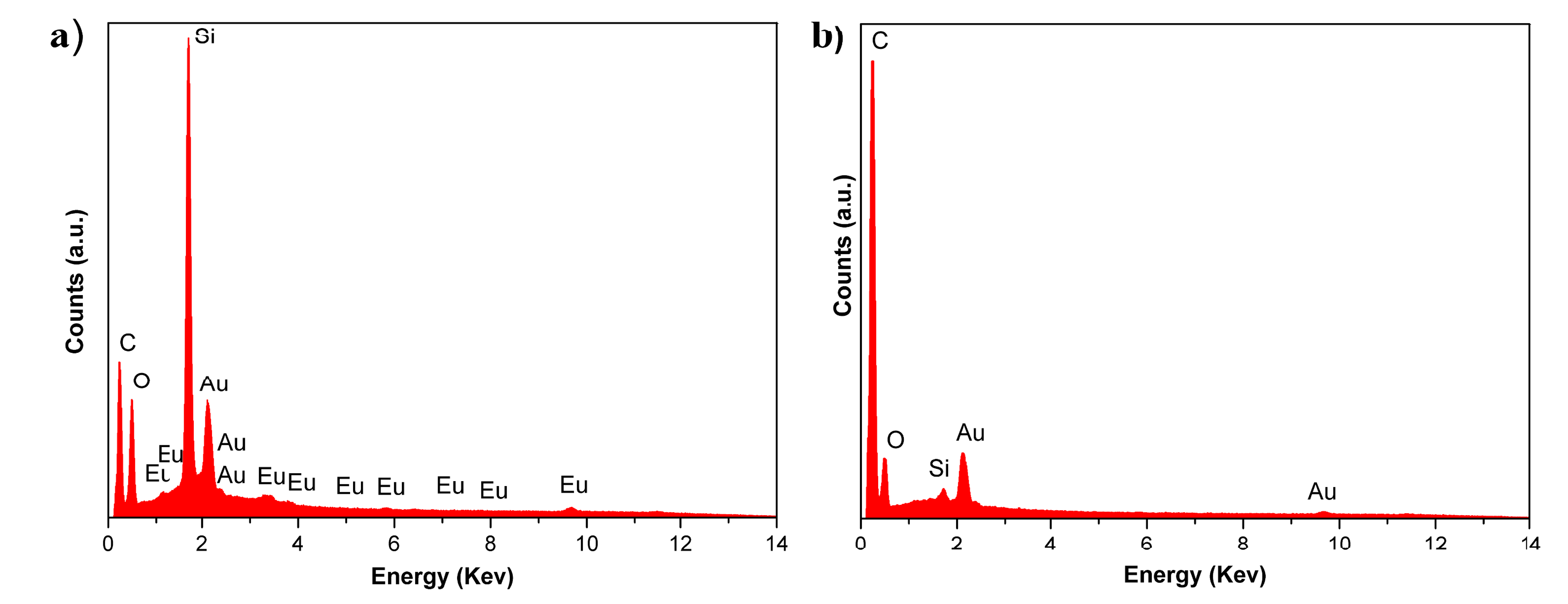

Supplement: S1 Fig — EDS spectra of EuBDC@AC inside (a) and outside of porous (b). (TIF) [file pone.0170026.s001.tif]

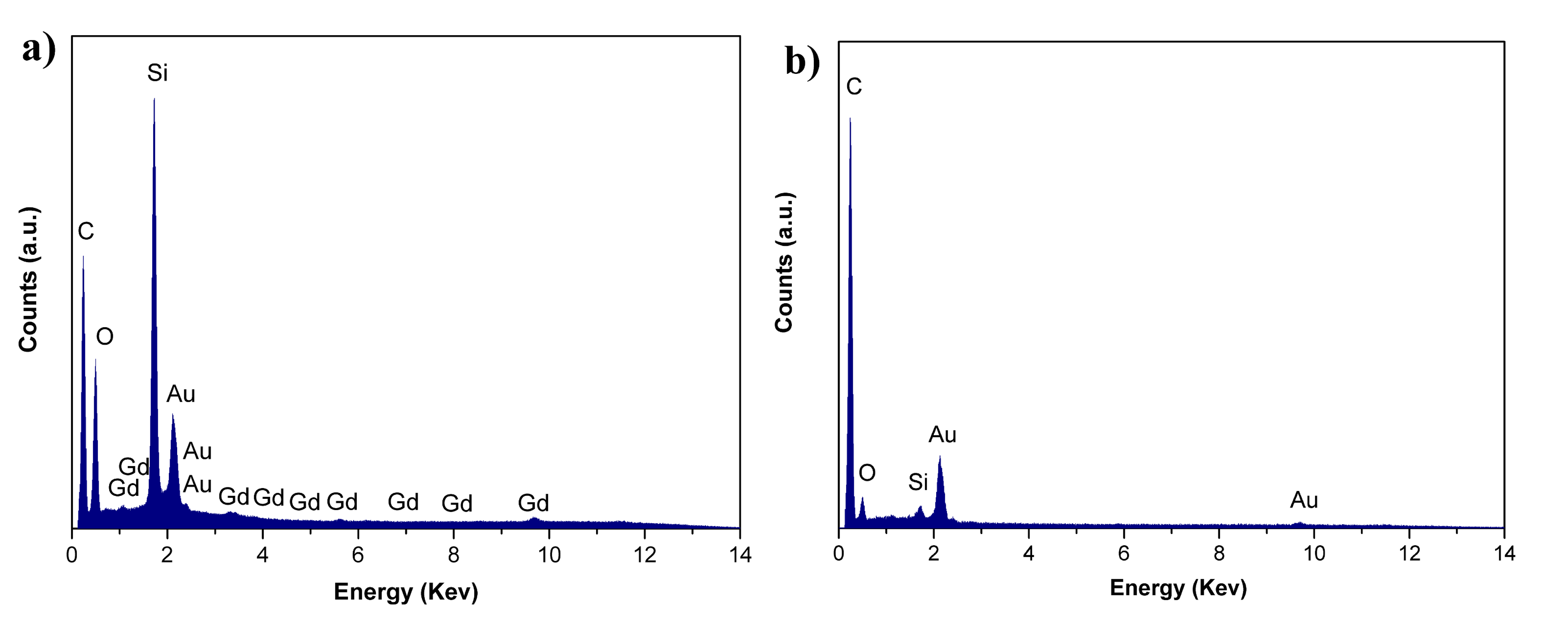

Supplement: S2 Fig — EDS spectra of GdBDC@AC inside (a) and outside of porous (b). (TIF) [file pone.0170026.s002.tif]

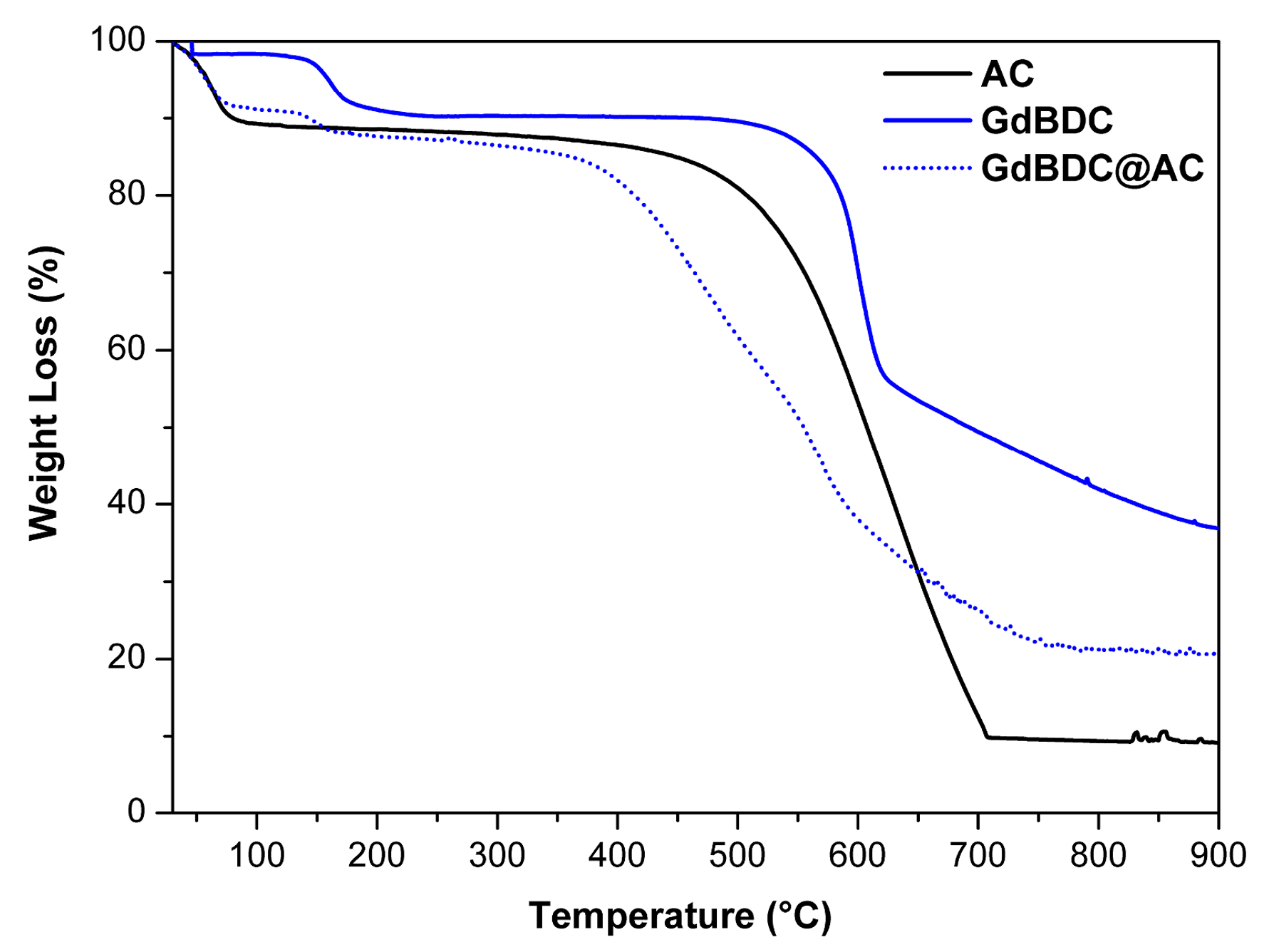

Supplement: S5 Fig — (TIF) [file pone.0170026.s005.tif]

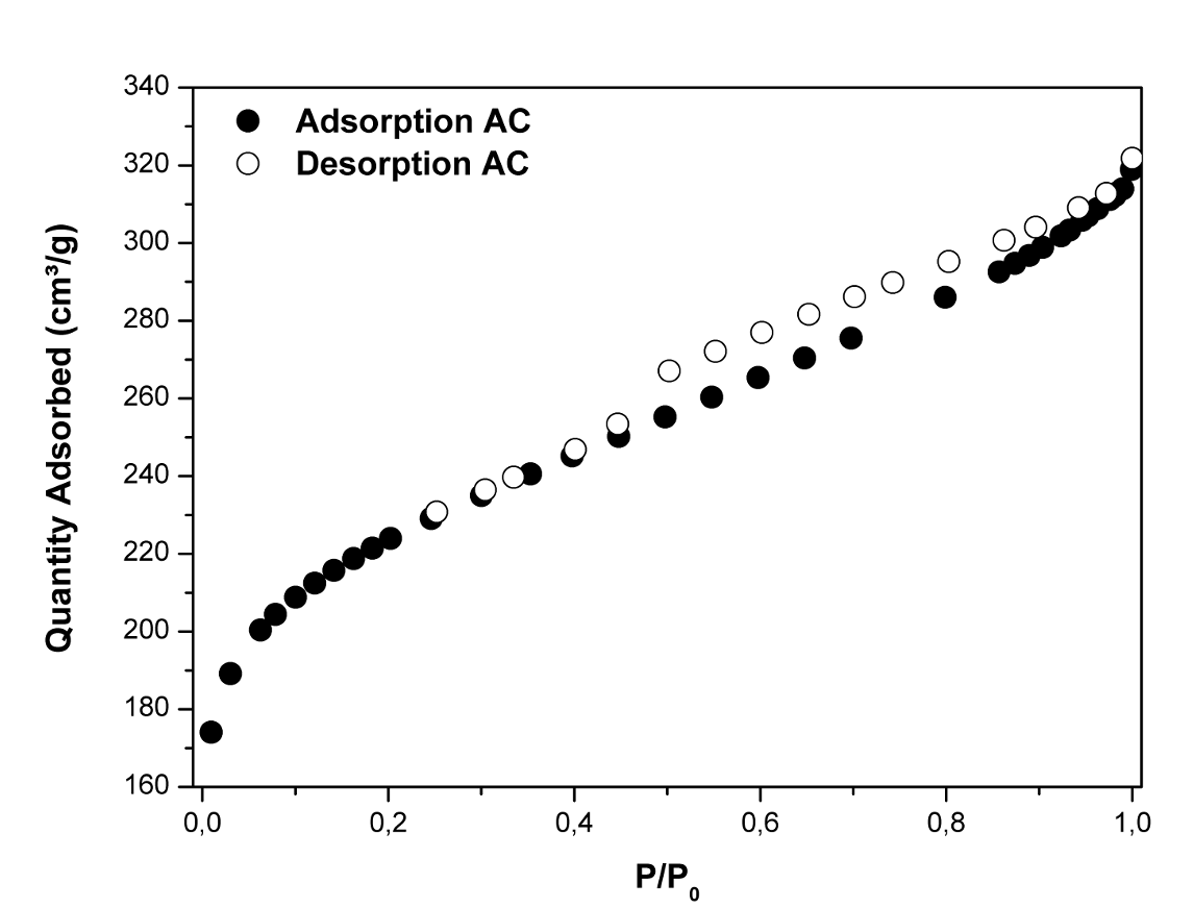

Supplement: S6 Fig — (TIF) [file pone.0170026.s006.tif]

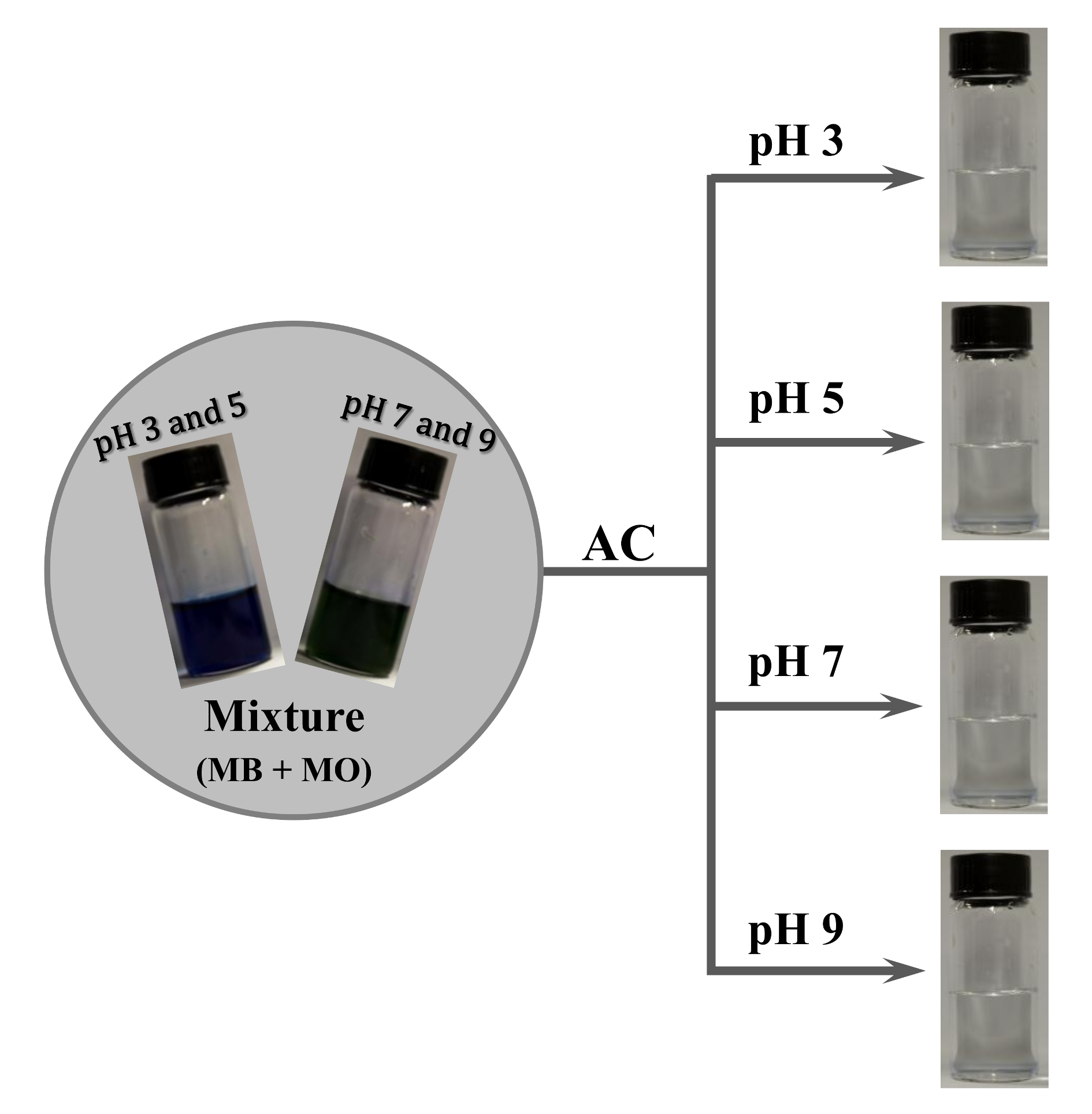

Supplement: S7 Fig — (TIFF) [file pone.0170026.s007.tiff]

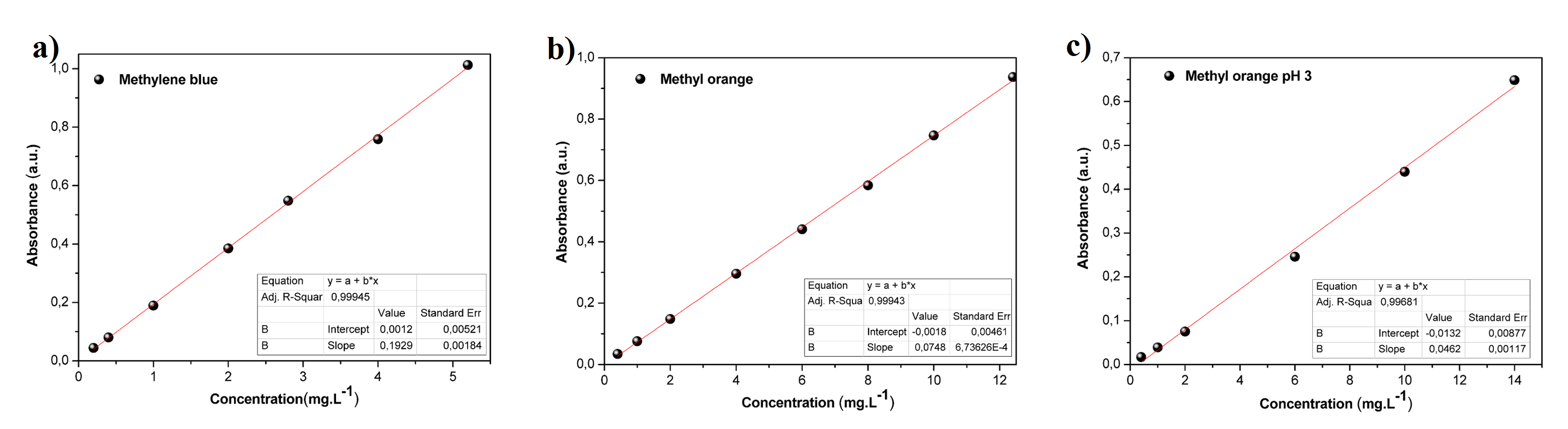

Supplement: S23 Fig — Calibrations curves of methylene blue in pH 7 (a), methyl orange in pH 7 (b) and methyl orange pH 3(c). (TIFF) [file pone.0170026.s023.tiff]
